# Effects of anthropogenic habitat disturbance and *Giardia duodenalis* infection on a sentinel species' gut bacteria

**DOI:** 10.1002/ece3.6910

**Published:** 2020-12-12

**Authors:** Sahana Kuthyar, Martin M. Kowalewski, Dawn M. Roellig, Elizabeth K. Mallott, Yan Zeng, Thomas R. Gillespie, Katherine R. Amato

**Affiliations:** ^1^ Department of Anthropology Northwestern University Evanston IL USA; ^2^ Departments of Environmental Sciences and Environmental Health and Program in Population Biology, Ecology, and Evolutionary Biology Emory University Atlanta GA USA; ^3^ Estación Biológica Corrientes Museo Argentino de Ciencias Naturales “Bernardino Rivadavia” (MACN‐CONICET) Corrientes Argentina; ^4^ National Center for Emerging and Zoonotic Infectious Diseases Centers for Disease Control and Prevention (CDC) Atlanta GA USA

**Keywords:** Alouatta, ecological interactions, microbiome, parasite, primate conservation

## Abstract

Habitat disturbance, a common consequence of anthropogenic land use practices, creates human–animal interfaces where humans, wildlife, and domestic species can interact. These altered habitats can influence host–microbe dynamics, leading to potential downstream effects on host physiology and health. Here, we explored the effect of ecological overlap with humans and domestic species and infection with the protozoan parasite *Giardia duodenalis* on the bacteria of black and gold howler monkeys (*Alouatta caraya*), a key sentinel species, in northeastern Argentina. Fecal samples were screened for *Giardia duodenalis* infection using a nested PCR reaction, and the gut bacterial community was characterized using 16S rRNA gene amplicon sequencing. Habitat type was correlated with variation in *A. caraya* gut bacterial community composition but did not affect gut bacterial diversity. *Giardia* presence did not have a universal effect on *A. caraya* gut bacteria across habitats, perhaps due to the high infection prevalence across all habitats. However, some bacterial taxa were found to vary with *Giardia* infection. While *A. caraya's* behavioral plasticity and dietary flexibility allow them to exploit a range of habitat conditions, habitats are generally becoming more anthropogenically disturbed and, thus, less hospitable. Alterations in gut bacterial community dynamics are one possible indicator of negative health outcomes for *A. caraya* in these environments, since changes in host–microbe relationships due to stressors from habitat disturbance may lead to negative repercussions for host health. These dynamics are likely relevant for understanding organism responses to environmental change in other mammals.

## Graphical abstract

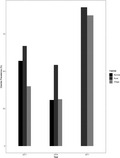

## 1 INTRODUCTION

Habitat disturbance, a common consequence of anthropogenic land use practices, can reduce survival and reproductive rates in some mammals, negatively affecting biodiversity (Arroyo‐Rodríguez & Mandujano, 2006; Barelli et al., 2015; Oklander et al., 2010). Traditionally, these effects have been linked to alterations in factors such as food availability and exposure to stressors, which can have direct physiological consequences (Chapman et al., 2006). Anthropogenically sourced habitat disturbance is also frequently associated with greater ecological overlap between humans, wild animals, and domestic species, increasing the potential for disease transmission from humans and domestic species to vulnerable wildlife (Faust et al., 2018; Goldberg et al., 2008). More recently, research suggests that these factors can influence mammalian physiology via interactions with the gut microbiome (Stumpf et al., 2016). For example, variation in diet across habitats are associated with differences in the gut microbiome of wild animals (Amato et al., 2013; Benítez‐Malvido & Martínez‐Ramos, 2003; Greene et al., 2019). Interactions among several host species within a shared environment may also facilitate the transmission of both commensal and pathogenic microbes, thus modifying a host's microbial community structure (Gomez et al., 2015; Moeller et al., 2013; Rwego et al., 2008oldberg, 2008). For example, humans, livestock, and nonhuman primates living in fragmented forests in Uganda share similar strains of *Escherichia coli*, highlighting the potential for bacterial transmission in sympatric environments (Rwego et al., 2008). Given that the gut microbiome is known to affect host nutrition, metabolism, immune function, and behavior (Flint et al., 2012; Heijtz et al., 2011; Hooper & Macpherson, 2010), changes to its structure as a result of any of these pathways are likely to have substantial impact on host physiology and ultimately, reproductive success and survival.

In the context of disturbance‐induced ecological overlap, the relationship between gut bacteria and pathogens is of particular interest. Gut bacteria may affect host susceptibility to intestinal pathogen infection and influence the progress of pathogenic infection and clinical manifestation of disease (Berrilli et al., 2012; Costello et al., 2012; Koch & Schmid‐Hempel, 2011; Partida‐Rodríguez et al., 2017). Alternatively, parasites may alter gut bacterial community composition, which could lead to alterations in host health (Barash et al., 2017; Berrilli et al., 2012; Cantacessi et al., 2014; Peachey et al., 2017; Šlapeta et al., 2015). There is limited research on the interactions between gastrointestinal parasites and gut bacteria and, of those, many focus on mice, amphibians, or humans (Barash et al., 2017; Berrilli et al., 2012; Cantacessi et al., 2014; Cooper et al., 2013; Jani & Briggs, 2014; Kreisinger et al., 2015; Lee et al., 2014; Shu et al., 2019). However, overall, the literature suggests that host–parasite–gut bacteria interactions are system‐specific. For example, increased microbial diversity is generally believed to indicate good host health (Costello et al., 2012). *Cryptosporidium* spp. infection in captive Coquerel's sifaka has been associated with decreased gut microbial diversity as well as bacterial taxa linked to dysbiosis (McKenney et al., 2017). In contrast, domestic cats infected with the protozoan *Giardia duodenalis* have higher microbial species richness compared to uninfected individuals (Šlapeta et al., 2015). Whether changes in microbial diversity due to pathogenic infection result in changes in host health and phenotype remain to be seen.

Given that habitat disturbance often also affects parasite prevalence and abundance patterns in wild mammals (Gillespie & Chapman, 2006, 2008; Gillespie et al., 2005; Zommerset al., 2013), parasite–bacteria relationships may be a key factor for understanding mammalian health outcomes in anthropogenically disturbed habitats (Cantacessi et al., 2014; Cooper et al., 2013; Kreisinger et al., 2015; Lee et al., 2014; Zaiss & Harris, 2016). Habitat disturbance may impact interactions between hosts and their associated microbial communities, which may then lead to downstream effects on host physiology and health, including nutritional deficits, higher prevalence of pathogens, and lower gut microbial diversity (Amato et al., 2013; Barelli et al., 2015; Estrada et al., 2017).

To improve our understanding of this interplay, we used the black and gold howler monkey, *Alouatta caraya*, as a model for exploring host–parasite–gut bacteria interactions in response to habitat disturbance. Primates are a relevant model in which to address these questions due to their large variation in habitat use, diet ecology, and interspecies interactions. As the most abundant primate species in northeastern Argentina, *A. caraya* is simultaneously a sentinel of ecosystem health and a model organism (Kowalewski & Gillespie, 2008, 2018; Kowalewski et al., 2011). For example, *A. caraya* experience high morbidity and mortality associated with yellow fever and thus serve as an early warning system prior to human outbreaks (Holzmann et al., 2010; Oklander et al., 2017).

Anthropogenic activities in Argentina, such as deforestation and selective logging, are forcing *A. caraya* into ecological overlap with humans and domestic animals. In such disturbed habitats, *A. caraya* interact with humans and domestic animals in multiple ways, including crossing terrestrially from forest patch to patch, sharing the same water sources as cattle, and engaging in altercations with domestic dogs (Kowalewski et al., 2011; Raño et al., 2016). These interactions may lead to greater susceptibility to zoonotic diseases and greater sensitivity to gut dysbiosis via horizontal microbial transmission.

We examined the effect of ecological overlap with humans and domestic species—a proxy for anthropogenic habitat disturbance—and infection by the protozoan parasite *Giardia duodenalis* on the gut bacteria of *A. caraya*. A previous study by Kowalewski and colleagues found a high prevalence of *G. duodenalis* in *A. caraya* populations across a gradient of disturbance (Kowalewski et al., 2011). As a result, we hypothesized that *A. caraya* in more disturbed habitats, with increased contact with humans and domestic animals, would have a higher infection prevalence of *G. duodenalis*.

Additionally, research across animals, including amphibians, fish, and other species of *Alouatta*, indicate that habitat disturbance is one factor that is associated with differences in gut bacterial community composition (Amato, 2016; Amato et al., 2013; Huang et al., 2018; Sullam et al., 2012). Therefore, we hypothesized that anthropogenic disturbance would be associated with differences in the *A. caraya* gut bacterial community. Due to stressors that accompany habitat disturbance, such as changes in diet or decreased home range (Chapman et al., 2006), we predicted A. *caraya* in more disturbed habitats would have decreased gut bacterial diversity.

Finally, as clinical manifestations of *G. duodenalis* have not yet been observed for *A. caraya* and given reported interactions between parasites and gut bacteria (Barash et al., 2017; Berrilli et al., 2012; Cantacessi et al., 2014; Cooper et al., 2013; Jani & Briggs, 2014; Kreisinger et al., 2015; Lee et al., 2014; Shu et al., 2019), it is crucial to understand how *G. duodenalis* affects the *A. caraya* gut bacteria and potentially *A. caraya* health. We hypothesized that *A. caraya* infected with *Giardia* would harbor a different gut bacterial community than uninfected individuals. In particular, based on studies of other primates (McKenna et al., 2008; McKenney et al., 2017), we predicted that *Giardia* infection in *A. caraya* would be correlated with decreased bacterial diversity and changes in bacterial community composition.

## 2 MATERIALS AND METHODS

### 2.1 Study site

This study was conducted in the winter (June–July) of 2016 and 2017 across three sites in the Corrientes and Chaco provinces in northeastern Argentina (Table 1): San Cayetano (27°34′S, 58°42′W), the Estación Biológica de Corrientes and its surroundings (27°30′S, 58°41′W), and Isla Brasilera (27°20′S, 58°40′W). Each *A. caraya* group, composed of three to 21 individuals, varied in their level of interaction with humans and domestic animals, as previously categorized by Kowalewski et al. (2011). San Cayetano is a village habitat where *A. caraya* share environments with humans and dogs. Isla Brasilera is a remote habitat where *A. caraya* are mostly isolated in a flooded forest. The Estación Biológica de Corrientes and its surrounding areas are classified as rural habitats where *A. caraya* share environments with cattle and are characterized by a semideciduous forest in a matrix of grassland that is vulnerable to deforestation (Kowalewski et al., 2011). All habitats are prone to flooding, and flash floods have been occurring more frequently in recent years, which could affect parasite prevalence and transmission. Notably, in April–May 2017, prior to our second period of sample collection, 600 mm of rainfall across 3 days was recorded, leading to severe flooding.

**Table 1:** Sampling sites

Site	Habitat characterization	Human‐associated species that share the environment
San Cayetano	Village	Humans and dogs
Estación Biológica de Corrientes and surrounding areas	Rural	Cows
Isla Brasilera	Remote	None
Cerrito	Village	Humans and dogs

### 2.2 Sample collection

Fecal samples were collected from 61 individuals (remote = eight individuals, rural = 29 individuals, village = 24 individuals) noninvasively and opportunistically during the 2016 and 2017 winter seasons, following previous protocols (Gillespie, 2006). Sex, age class (infant, juvenile, subadult, or adult), social group, and habitat type of each individual were noted (Table S1). For the most part, social groups, and thus individuals, were sampled only once during the study. We sampled between five and seven social groups per site, and almost all individuals from each social group were sampled and sequenced. Individuals were sampled for both *Giardia* presence and gut bacterial community characterization. For each sample, one gram of fecal matter was immediately homogenized in sterile cryovials in RNAlater nucleic acid stabilizing buffer (Ambion, Life Technologies, Grand Island, NY) for *Giardia* analysis, and another gram was immediately homogenized in sterile fecal vials with 95% ethanol for microbiome analysis. All samples were stored at 4°C until transport to the USA for processing, and all research procedures were approved by Emory University and Northwestern University (Northwestern IACUC Field Research 2019‐001) and complied with applicable laws in Argentina. Import and export permits were obtained from the Centers for Disease Control (CDC) and Argentina's Ministerio de Ambiente y Desarrollo Sostenible, respectively.

### 2.3 Giardia duodenalis analyses

All *Giardia* analyses were conducted at Emory University, Atlanta, Georgia. The CDC provided technical assistance with all *Giardia* protocols. Due to the heterogeneity of *G. duodenalis*, a multilocus approach was utilized to target three genes: glutamate dehydrogenase (*gdh*), beta‐giardin (*bg*), and triosephosphate isomerase (*tpi*). DNA was extracted from the RNAlater‐preserved fecal samples using the FastDNA Spin Kit for Soil (MP Biomedicals LLC), and the multilocus genes were amplified using a nested PCR protocol adapted from Roellig et al. (2015). Briefly, all PCR reactions were prepared in a final volume of 25 μl containing 1× Taq PCR Master Mix (Qiagen), 400 ng/ μl BSA, 500 nM of each primer, nuclease‐free water, and genomic DNA (2 μl in first PCR reaction and 2 μl of first reaction product in the second PCR reaction) (amplification conditions listed in Table S2). Positive and negative controls were included in each reaction. The presence of *Giardia* infection was verified by running 5 μl of PCR product on a 1% agarose gel.

### 2.4 Microbiome analyses

DNA was extracted from the ethanol‐preserved fecal samples using the Qiagen Powersoil Kit at Northwestern University, Evanston, Illinois. The V4 region of the 16S ribosomal RNA gene was amplified using a modified version of the Earth Microbiome Project protocol (Mallott & Amato, 2018; Thompson et al., 2017) and the 515Fa/926R primer set (Walters et al., 2016). Extraction and negative controls were both included in the sequencing run. We barcoded and pooled all amplicons in equimolar concentrations for sequencing on the Illumina MiSeq V2 platform at the DNA Services Facility at the University of Illinois at Chicago.

Paired‐end sequences were joined and processed using QIIME2 v2019.7 (Bolyen et al., 2019). Quality filtering and the removal of chloroplast and mitochondria sequences resulted in a total of 856,786 reads with an average of 13,387 reads per samples. The DADA2 algorithm was used to cluster amplicon sequence variants (ASVs), and taxonomy was assigned by comparing ASVs to the GreenGenes 13.8 reference database. When we compared taxonomic assignments between Greengenes and SILVA, we found that the Greengenes database assigned more features (2,568) than SILVA (2,559) did for this particular dataset. All samples were rarefied to 8,000 reads per sample based on alpha rarefaction curves (Figure S1a–c). Alpha diversity (Faith's phylogenetic distance, Shannon diversity index, and number of observed ASVs) and beta diversity (unweighted and weighted UniFrac distances) were calculated in QIIME2.

### 2.5 Statistical analyses

For *Giardia* analyses, infection prevalence per site was calculated as the proportion of individuals infected divided by the number of individuals sampled per site. Chi‐square tests of independence were utilized to test whether infection prevalence differed significantly across sites, with a *p*‐value cutoff of .05. All statistical analyses for microbiome data were performed with *p*‐values cutoffs at both .001 and .05. Permutational analyses of variance (PERMANOVA) using the *adonis* function (R Core Team, 2014) from the *vegan* package in R (Oksanen et al., 2008) were utilized to test for significant differences in bacterial community composition across habitats using both unweighted and weighted UniFrac distances. Only eight samples from remote habitats were collected (due to flooding), so for subsequent analyses on alpha diversity and linear mixed effects (LME) models, we filtered the dataset to include only samples from rural and village habitats and tested for differences due to habitat, group, year, and *Giardia* presence/absence, while controlling for individual identity. The *nlme* package in R (Pinheiro & Bates, 2010) was used to run linear mixed effects models to examine the effects of habitat and *Giardia* presence, separately, on alpha diversity indices and the relative abundance of gut bacteria at the genus level, while controlling for individual identity. Finally, linear discriminant analysis of effect size (LEfSe) through a Galaxy server was utilized to understand which bacterial taxa discriminated habitat type and infection status (Afgan et al., 2018; Segata et al., 2011).

## 3 RESULTS

### 3.1 Giardia infection

Total *Giardia* prevalence was 82.6% (52/63 individuals) across all habitats. Almost all individuals were infected with *G. duodenalis* in rural (87.9% (26/29 individuals)) and village (88.5% (23/26 individuals)) habitats, yet only 37.5% (3/8) of *A. caraya* were found to be infected with *Giardia* in remote habitats. There was no significant difference in infection prevalence between *A. caraya* in rural and village habitats. However, the differences in infection were significant between *A. caraya* in remote and rural habitats (*x* ^2^ = 10.06, *df* = 1, *p*‐value < .05) as well as those between individuals in remote and village habitats (*x* ^2^ = 8.83, *df* = 1, *p*‐value < .05).

**Figure 1:**
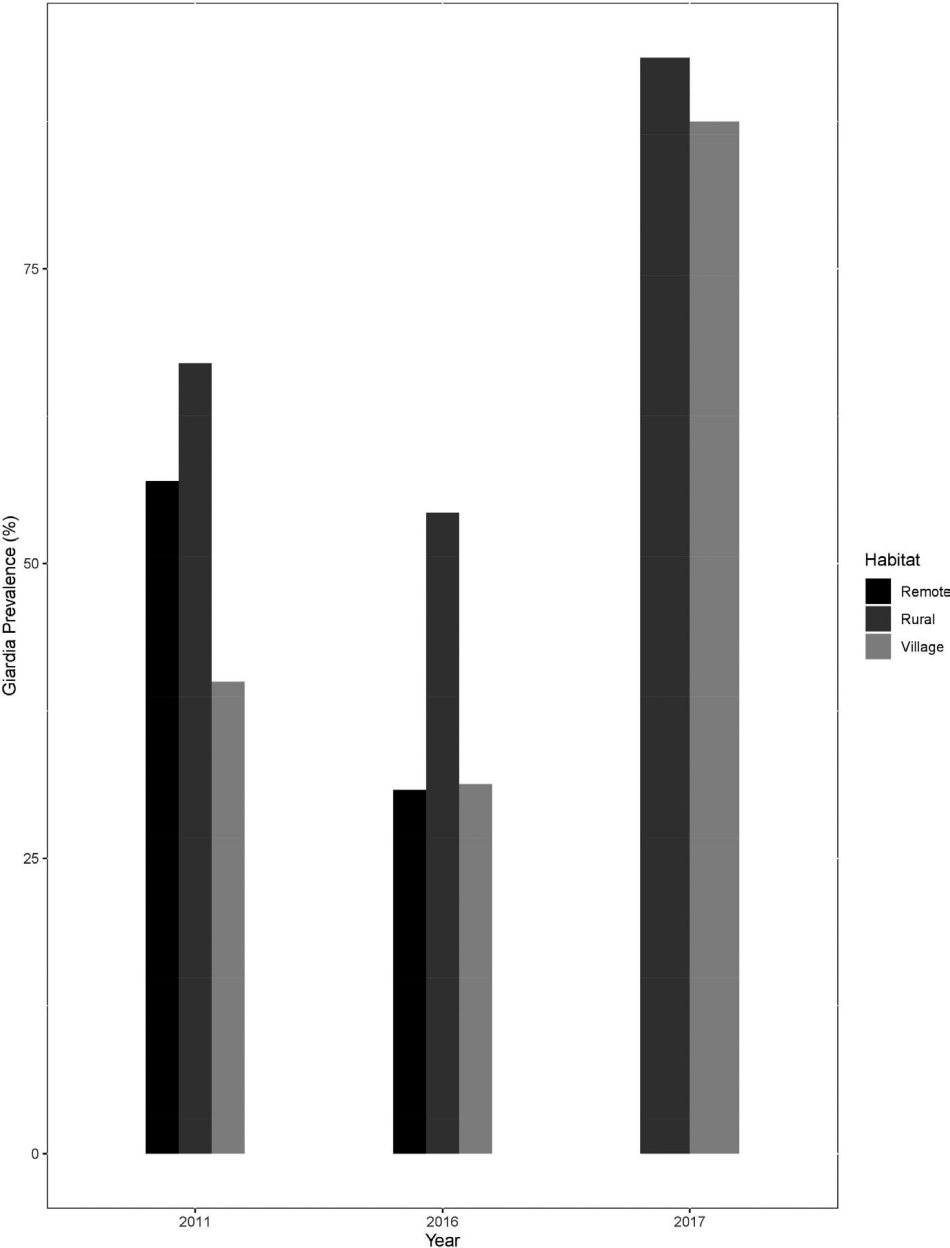
*Giardia duodenalis* prevalence in *Alouatta caraya* across years and habitats

We previously screened for *Giardia* infection in this *A. caraya* population in 2011 and 2016 for a different study, and prevalence in both rural and village habitats was significantly lower (rural, = 11.97, *df* = 1, *p*‐value < .05; village, = 19.2, *df* = 1, *p*‐value < 0.05) during these years compared to our data from 2017 (Figure 1) (Kowalewski et al., 2011; Kuthyar, unpublished data).

### 3.2 Habitat disturbance and gut bacteria

**Figure 2:**
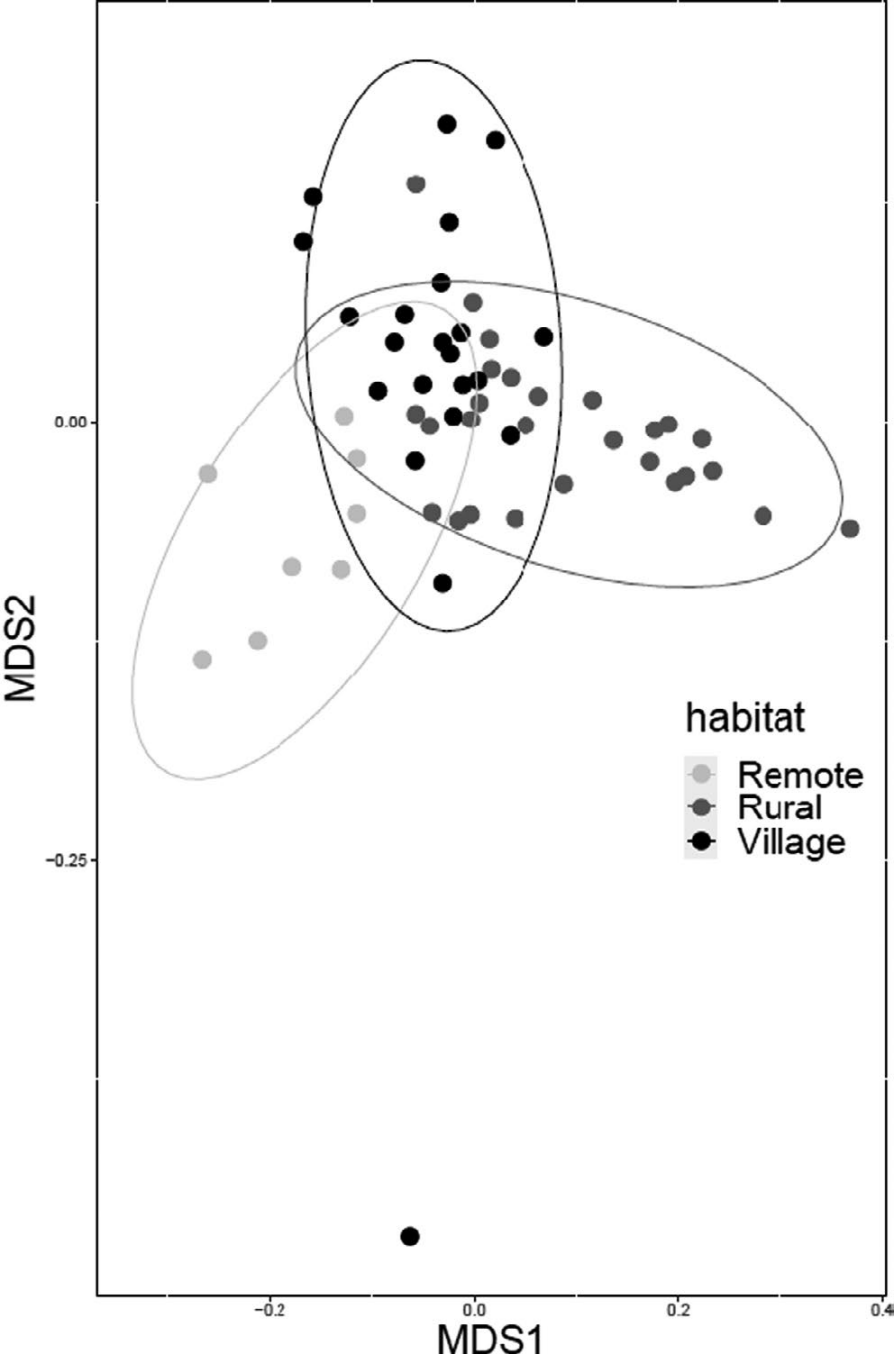
Beta diversity across habitats based on the weighted UniFrac distance matrix

**Figure 3:**
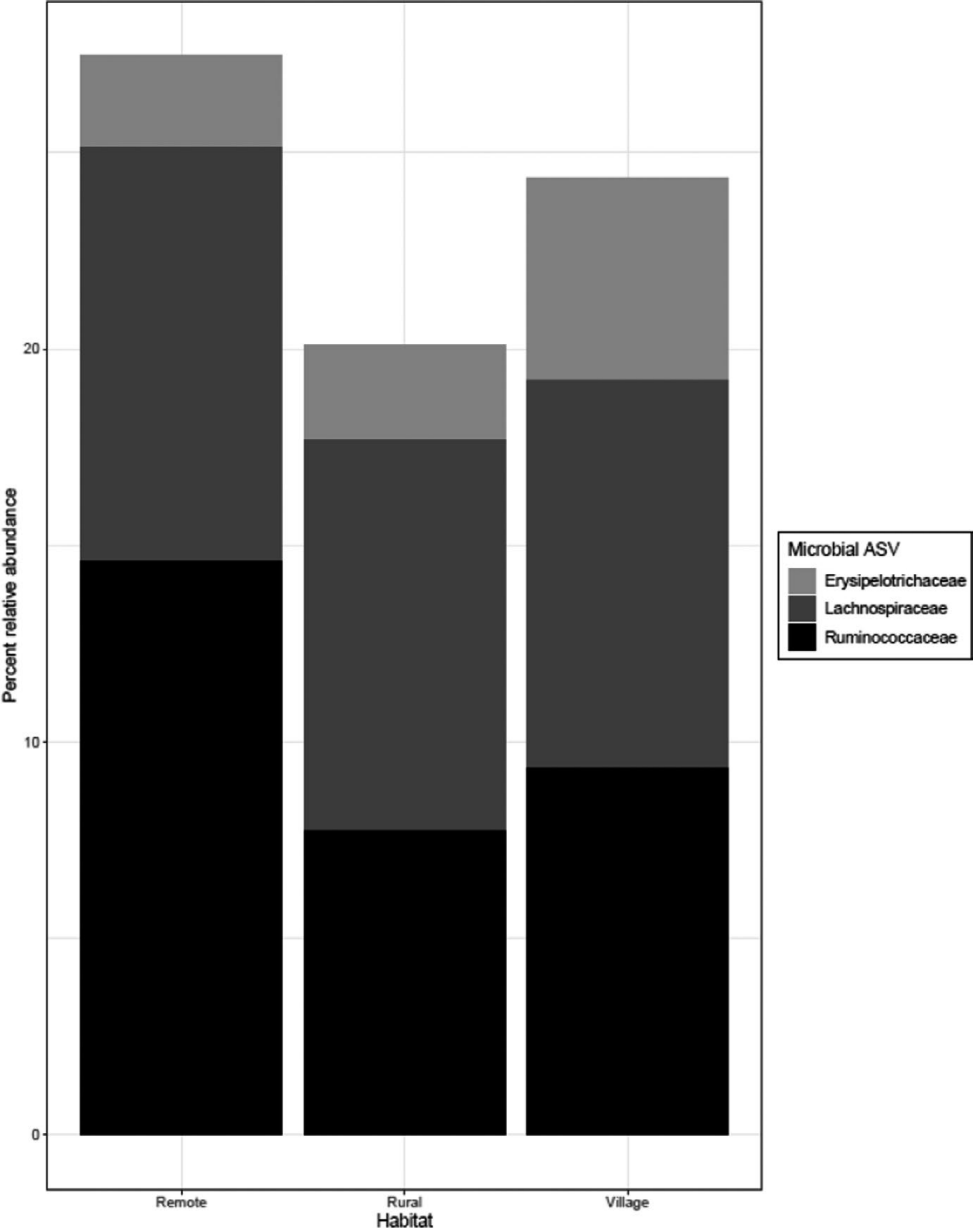
Average relative abundance of bacterial taxa significantly differentially expressed (LDA > 2) in *Alouatta caraya* between rural and village habitats

Bacterial community composition varied across habitats (Figure 2; unweighted UniFrac: PERMANOVA *F*_2,38_ = 3.22, *R* ^2^ = 0.0963, *p*‐value < .001, weighted UniFrac: PERMANOVA *F*_2,38_ = 10.4537, *R* ^2^ = 0.238, *p*‐value < .001) and social groups (unweighted UniFrac: PERMANOVA *F*_15,38_ = 1.37, *R* ^2^ = 0.307, *p*‐value < .001, weighted UniFrac: PERMANOVA *F*_15,38_ = 1.80, *R* ^2^ = 0.307, *p*‐value < .001). There were no significant results when we ran a model with an interaction between habitat type and *Giardia* presence. Further, there was no difference in any of the richness or diversity indices with respect to habitat type or social group (all *p* > .05). However, LEfSe identified several bacterial taxa that were differentially abundant at the family level (Figure 3) and at the ASV level. We found *A. caraya* in rural habitats were enriched with *Erysipelotrichaceae, Lachnospiraceae,* and *Bacteroides uniformis,* whereas *A. caraya* in village habitats were enriched with *Ruminococcaceae* and *Prevotella copri*.

### 3.3 Giardia infection and gut bacteria

**Figure 4:**
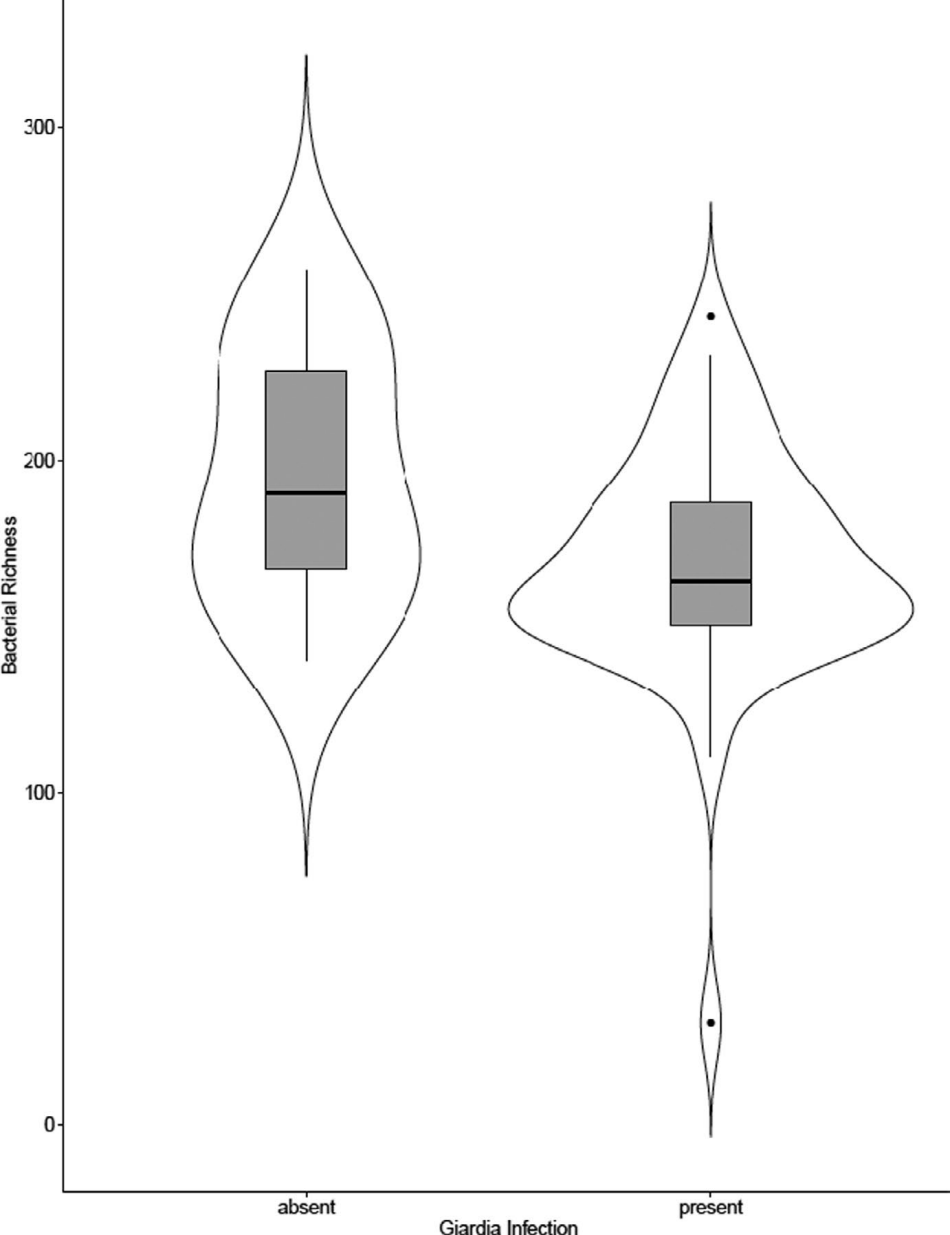
Significant differences in bacterial richness (observed ASVs) between Giardia‐infected and ‐uninfected *Alouatta caraya* individuals

Despite the high prevalence of *Giardia*, infection was not associated with variation in overall gut bacterial community composition within or across habitats (all *p*‐value > .05). Further, Shannon diversity (measuring abundance and evenness of the microbial species present) and Faith's phylogenetic distance (measuring the phylogenetic diversity of the microbial community) did not correlate with *Giardia* infection. However, bacterial richness (observed ASVs) varied significantly with *Giardia* infection (*F*_1,46_ = 8.42, *p*‐value < .05). On average, there were less observed ASVs in *Giardia*‐infected individuals when compared with uninfected individuals (Figure 4). Additionally, the relative abundance of nine out of 68 taxa at the family level was significantly associated with *Giardia* presence (q‐value < 0.05), including *Lachnospiraceae, Clostridiales, Cyanobacteria, Anaeroplasmataceae, Dethiosulfovibrionaceae, Ruminococcaceae, Mollicutes*, and *Coriobacteriaceae*. Finally, LEfSe analysis at the ASV level showed that there were 19 bacterial taxa that were differentially abundant in uninfected individuals (Table S3), following the trend that uninfected individuals may harbor more distinct ASVs than infected individuals.

## 4 DISCUSSION

This study examined the effects of habitat disturbance and *G. duodenalis* infection on the *A. caraya* gut microbiota in northeastern Argentina. As hypothesized, we found that habitat type was significantly associated with differences in both *Giardia* infection prevalence and gut bacterial community composition. There was also a significant interaction of *Giardia* infection with the relative abundance of specific bacterial taxa and bacterial richness. These results suggest that examinations of gut bacteria‐parasite interactions are important to include in studies of host physiological responses to environmental change.

### 4.1 Habitat disturbance and Giardia duodenalis

Habitat disturbance leads to increased contact between wildlife, livestock, and humans, which increases the risk for zoonotic disease transmission. In our study, habitat differences in ecological overlap were associated with differences in *G. duodenalis* prevalence. *Giardia duodenalis* prevalence in *A. caraya* was higher in rural and village habitats compared to remote habitats. This pattern may be a result of increased contact with humans and livestock. *Alouatta caraya* share environments with cattle in rural habitats and with humans and dogs in village habitats, whereas they are mostly isolated in remote habitats. This variation in interaction may put *A. caraya* in rural and village habitats at higher risk for cross‐species transmission of *Giardia* spp. and other parasites. Further, *A. caraya* in rural habitats display higher rates of terrestriality due to habitat fragmentation, as individuals cross on the ground between patches of forest, leading to increased interaction with both animals and infective parasite cysts and thus, a higher frequency of infection.

In these rural and village habitats, we also detected a difference in *G. duodenalis* prevalence across years. Due to a massive flood in April 2017, we were not able to collect samples from remote habitats that year, limiting our sample size for comparison. However, when we compared our data to results from previous years, *Giardia* prevalence in both rural and village habitats was much higher in 2017 than in other years. Although previous studies have found gastrointestinal parasitic infection in howler monkeys living in disturbed habitats (Eckert et al., 2006; Trejo‐Macías & Estrada, 2012; Trejo‐Macías et al., 2007; Vitazkova & Wade, 2006), the high infection rates found in rural and village habitats in 2017 most likely resulted from water contamination from the 2017 flooding event, as all sampling sites were inundated. High humidity favors the survival of the infectious stages of *Giardia* cysts (Martíez‐Mota et al., 2015), and parasite prevalence has been associated with high levels of precipitation in *A. caraya* habitats (Kowalewski & Gillespie, 2008). *Giardia* cysts are also more infectious for a longer period of time in water than in soil and feces (Olson et al., 2010), so flooding may have spread infective *Giardia* cysts across rural and village habitats. As climate change and anthropogenic disturbance is increasingly impacting the landscape and forest fragmentation, cycles of flooding have changed from every 15 years to every 2 or 3 years. The increased *Giardia* prevalence in 2017 may reflect this change in the flooding cycle and may predict future alterations in *Giardia* dynamics in the ecosystem.

### 4.2 Habitat disturbance and gut bacteria

Habitat type was also associated with variation in the *A. caraya* gut bacterial community, where different habitats were associated with different gut bacterial community compositions. Multiple factors may contribute to these patterns, including diet, intra‐ and interspecies contact, and the physical environment (Mccord et al., 2014).

Several bacterial taxa varied between individuals living in rural and village habitats, including *Lachnospiraceae*, *Erysipelotrichaceae, Ruminococcaceae, Bacteroides uniformis*, and *Prevotella copri—*a pattern that may be associated with diet. Previous studies have shown that differences in diet composition, and thus nutrient availability, across habitats influence the types of gut bacteria residing in animal hosts (Amato et al., 2013; Barelli et al., 2015; Trosvik et al., 2018). The same bacterial taxa in which we detected variation in our study showed a response to changes in diet and/or habitat in other studies. High abundances of *Prevotella* have been found in vervets and macaques eating a Western diet (Amato et al., 2015; Ma et al., 2014), red colobus monkeys in protected forest in Tanzania (Barelli et al., 2015), and black howler monkeys consuming a leafy diet (Amato et al., 2014, 2019). *Ruminococcaceae* relative abundances are reported to be higher in red colobus monkeys in disturbed habitats (Barelli et al., 2015), in black howler monkeys during seasons of low energy intake (Amato et al., 2014), and in frogs with low dietary diversity in farmland habitats (Chang et al., 2016). Similar dynamics between these bacterial taxa and diet may be operating in our system. In *A. caraya*, there are differences in overall seasonal patterns of food availability as well as the availability of new and mature leaves across habitats (Kowalewski & Zunino, 2004), and lower food abundances have been recorded in rural and village habitats compared to remote habitats (Zunino et al., 2001). Since *Prevotella* spp. has been associated with metabolism of starches (Kovatcheva‐Datchary et al., 2009), the increased abundance of *Prevotella copri* in village habitats may be because individuals in these habitats sometimes receive fruit and bread from residents (M. Kowalewski, personal observation). These differences in food availability and consumption have the potential to affect competitive interactions between bacteria in the gut, thereby altering community composition overall. However, since we did not collect diet data in this study, it is unclear whether previously documented differences in food availability correlate to differences in actual food consumption across sites. Future studies should incorporate data on *A. caraya* diet to see which dietary factors (i.e., leaf and fruit abundance, rate human food scrap consumption, proportion of the diet that is fruit, or amounts and ratios of specific nutrients consumed) drive these changes in bacterial taxa across habitats.

Interestingly, there was no difference in bacterial richness or diversity across habitat types in *A. caraya*. This finding is in contrast to other studies in a range of animals. For example across various species of primates, including another species of howler monkey, habitat fragmentation has a modest but significant effect on gut bacterial diversity (Amato et al., 2013; Barelli et al., 2015; Mccord et al., 2014). Additionally, house sparrows in urban environments were characterized by decreased microbial diversity and fewer metabolic functions (Teyssier et al., 2018). These patterns are often attributed to variation in diet since diet diversity is often linked to gut microbiome diversity (Heiman & Greenway, 2016), and habitat disturbance often alters diet diversity as a function of a decline in plant species diversity in the habitat and food availability. Indeed, reduced dietary diversity is associated with reduced bacterial diversity in both red colobus monkeys and howler monkeys in fragmented and/or disturbed habitats (Amato et al., 2013; Barelli et al., 2015). However, habitat disturbance and fragmentation may not impact the interaction between diet and gut bacterial structure in the same way across all species. For example, vampire bats exhibit less diverse diets in fragmented habitats but show no changes in gut bacterial diversity between habitats (Ingala et al., 2019). Furthermore, in the disturbed rural and village habitats of this study, *A. caraya* diet composition may be more diverse compared to diets observed in remote habitats (M. Kowalewski, personal observation) as a result of edge effects and secondary forest succession after selective deforestation (Kowalewski & Zunino, 1999). Future research should include data on actual food consumption to further explore the interaction between diet diversity and gut microbial diversity.

Beyond diet, patterns of microbial transmission may also contribute to the cross‐habitat differences in bacterial community composition identified in this study. Evidence of these dynamics has been reported previously. For example, gorillas that lived in ecological overlap with humans and livestock in Rwanda harbored similar strains of *E. coli* to those of humans and livestock compared to gorillas that did not overlap (Rwego et al., 2008). Further, a study in Uganda found that *Cryptosporidium* spp. isolates looked genetically identical regardless if they came from a human, nonhuman primate, or livestock source (Salyer et al., 2012). Here, as high ecological overlap exists among *A. caraya*, humans, and domestic animals in the anthropogenically disturbed habitats, *A. caraya* in fragmented rural and village habitats may be more exposed to cross‐species bacterial transmission, thus altering their bacterial community structure, than at remote habitats, where they are mostly isolated from humans and livestock.

Finally, the physical environment and the resulting exposure to environmental bacterial pools may shape a host's gut bacteria across habitats. In a previous study, soil was found to best predict the gut bacteria of baboons in Kenya (Grieneisen et al., 2019). Since baboons are terrestrial and *A. caraya* are arboreal, soil itself may not have as strong an influence on the *A. caraya* gut bacterial community in our study. However, contact with other substrates in arboreal habitats could have a similar effect. Furthermore, some of the *A. caraya* groups sampled in this study are commonly reported to travel on the ground between patches of fragmented forest at rural sites (M. Kowalewski, personal observation). This behavior is likely to increase their contact with terrestrial substrates (e.g., soil and animal feces) and may lead to patterns of environmental bacterial acquisition similar to those observed in the baboons. Moving forward, we hope to measure soil samples to test this mechanism.

### 4.3 Gut bacteria and Giardia duodenalis

As hypothesized, *G. duodenalis* infection was associated with differences in gut bacterial community membership. Past studies in frogs found that parasitic infection led to altered skin bacterial communities and even increased the pathogen load in some cases (Jani & Briggs, 2014; Shu et al., 2019). Certain bacterial taxa, such as *Lachnospiraceae, Anaeroplasmataceae*, and *Ruminococcaceae*, that varied with *Giardia* infection are all associated with providing crucial metabolic services to the host (Biddle et al., 2013; Petzel et al., 1989), and changes in their abundance could have impacts on howler health beyond susceptibility to infection and symptoms of disease. The modified bacterial community structure, along with living in a disturbed habitat and harboring a parasite, could further alter the nutritional, and thus overall health, status of *A. caraya* in these contexts.

Bacterial richness was also significantly associated with infection, where infected individuals had fewer observed ASVs on average compared to uninfected individuals. It is possible these reductions in diversity could enhance the potential for colonization by potentially pathogenic microbes, such as *Enterococcus* sp. (Iebba et al., 2016). However, more research is necessary. Previously, parasite infection was associated with decreased microbial diversity in birds in the Galapagos, where initial infection led to changes in host behavior, leading to further susceptibility to future infection (Knutie, 2018). However, infection with chytrid fungus in frogs was associated with increased bacterial diversity (Becker et al., 2017). In past studies, *Giardia* has been associated with bacterial overgrowth as well as changes in the relative abundance of certain taxa, including increased abundance of *Proteobacteria* and decreased abundance of *Firmicutes* and *Bacteroidetes* (Barash et al., 2017; Halliez & Buret, 2013; Müller & Von Allmen, 2005). Additionally, dogs infected with *Giardia* had significant differences in their bacterial community structure compared to uninfected dogs (Šlapeta et al., 2015). The implications of these changes to host health both in the present study and across the literature remain to be seen.

It is also important to note *Giardia* infection did not have a universal effect on the *A. caraya* gut bacteria across habitats. Since *A. caraya* exhibited distinct gut bacterial communities across habitats, the composition of their microbiomes in each type of habitat could increase or decrease their susceptibility to *G. duodenalis*. Further, due to these differences in gut bacterial communities, host physiological responses to parasitic infections, such as *Giardia* and other gastrointestinal parasites, could vary with habitat type. The presence of infection may also impact host response to habitat disturbance as individuals infected with *Giardia* may or may not survive better in a given habitat depending on the parasite's effects on the gut bacterial community.

A number of factors constrained this study. Due to the flooding event in 2017, we were not able to collect fecal samples from *A. caraya* individuals living in the remote habitat, which limited the comparison of *Giardia* infection across habitats. Additionally, we did not record specific measures of host health, such as stress levels or immune function, which may have demonstrated whether changes in gut bacterial communities due to habitat disturbance and/or *Giardia* infection impacted *A. caraya* health. A more robust sample size both within habitats as well as across years would provide a more informed understanding of how habitat disturbance and *Giardia* infection affect the *A. caraya* gut microbiome. Further, since *G. duodenalis* is a multispecies complex with eight distinct genotypes and degrees of virulence (Ryan & Cacciò, 2013), future research should explore if different *Giardia* genotypes have different interactions with the gut microbiome and thus consequences for host health.

Overall, this study indicates that anthropogenic habitat disturbance influences multiple groups of mammalian gut microbes and that these microbes may also affect each other. Understanding the influences these interactions have on host health outcomes in disturbed habitats will be important for conservation efforts moving forward. Habitat disturbance may not only affect host health through shifts in food availability and quality, but also indirectly through changes to host‐associated bacterial communities and susceptibility to parasite infection. Further, integration of the gut microbiome into a disease ecology framework will be crucial in understanding the intrinsic and extrinsic factors that impact host survival and reproduction.

As a sentinel species of this semideciduous ecosystem, *A. caraya* plays a crucial role in advising wildlife health surveillance of increasingly fragmented and disturbed habitats. Their behavioral plasticity and dietary flexibility allow them to exploit a large range of habitat conditions, resulting in resilience in fragmented and disturbed habitats (Kowalewski et al., 2011; Miner et al., 2005). As habitats are becoming harsher due to anthropogenic pressures, however, previously resilient wild animals may be reaching their limits of plasticity. In addition to more obvious factors, the understudied interactions between habitat disturbance, pathogen transmission, and a host's gut bacteria could negatively influence host health, reproductive output, and even survival. As such, monitoring both the host's gut bacterial community and pathogen load could be an important noninvasive method to improve understanding of the effects of anthropogenic disturbance on wild mammal health and inform conservation strategies.

## CONFLICT OF INTEREST

None declared.

## AUTHOR CONTRIBUTIONS

**Sahana Kuthyar:** Conceptualization (lead); formal analysis (lead); funding acquisition (equal); investigation (lead); methodology (lead); writing–original draft (lead); writing–review and editing (equal). **Martin M. Kowalewski:** Conceptualization (equal); funding acquisition (supporting); investigation (equal); methodology (equal); resources (equal); supervision (equal); writing–review and editing (equal). **Dawn Roellig:** Formal analysis (supporting); methodology (supporting); resources (equal); supervision (supporting); writing–review and editing (equal). **Elizabeth K. Mallott:** Formal analysis (equal); investigation (equal); methodology (equal); software (equal); supervision (equal); writing–review and editing (equal). **Yan Zeng:** Formal analysis (supporting); methodology (supporting). **Thomas R. Gillespie:** Conceptualization (equal); formal analysis (equal); funding acquisition (equal); investigation (equal); methodology (equal); project administration (equal); resources (equal); software (equal); supervision (lead); writing–review and editing (equal). **Katherine R. Amato:** Conceptualization (equal); formal analysis (equal); funding acquisition (equal); investigation (equal); methodology (equal); project administration (equal); resources (equal); software (equal); supervision (lead); writing–review and editing (equal).

## Supporting information

Appendix S1

## Data Availability

Scripts for QIIME2 and R can be found at https://github.com/Kramato‐lab/kuthyar_giardia. The datasets used for analyses can be accessed via Dryad at https://doi.org/10.5061/dryad.wm37pvmj5. All sequences have been uploaded to SRA and will be publicly accessible with publication (PRJNA607274).
