# Supplementary material for: Effects of anthropogenic habitat disturbance and Giardia duodenalis infection on a sentinel species' gut bacteria
Source: Ecol Evol. 2020 Dec 12;11(1):45–57. doi: 10.1002/ece3.6910 (PMC7790644; doi:10.1002/ece3.6910)
Supplement: Supplementary file 1 — Appendix S1 [file ECE3-11-45-s001.docx]

**Supplementary Information**

*Supplementary Figure 1 (a-c)*, Alpha rarefaction curves based on the filtered relative abundance data. Figure 1a is based on Shannon diversity.


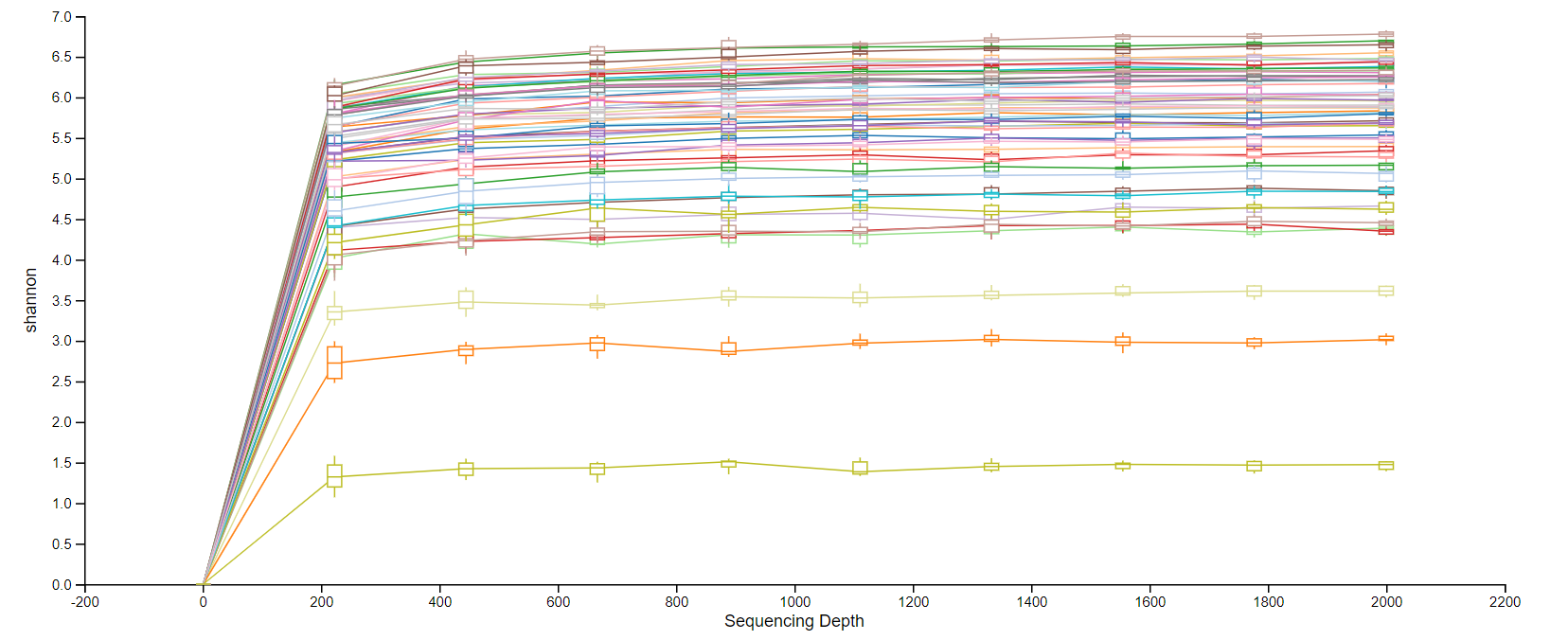


Figure 1b is based on Faith’s phylogenetic diversity.


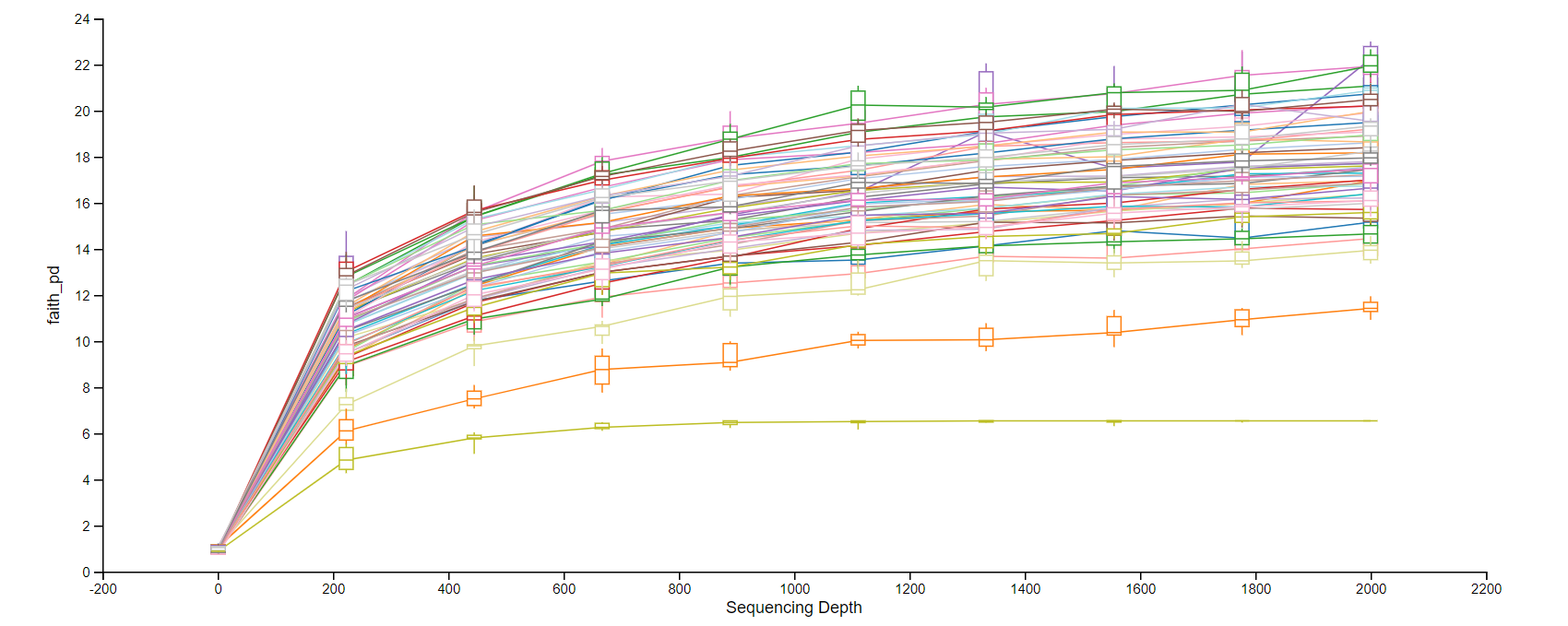


Figure 1c is based on observed OTUs.


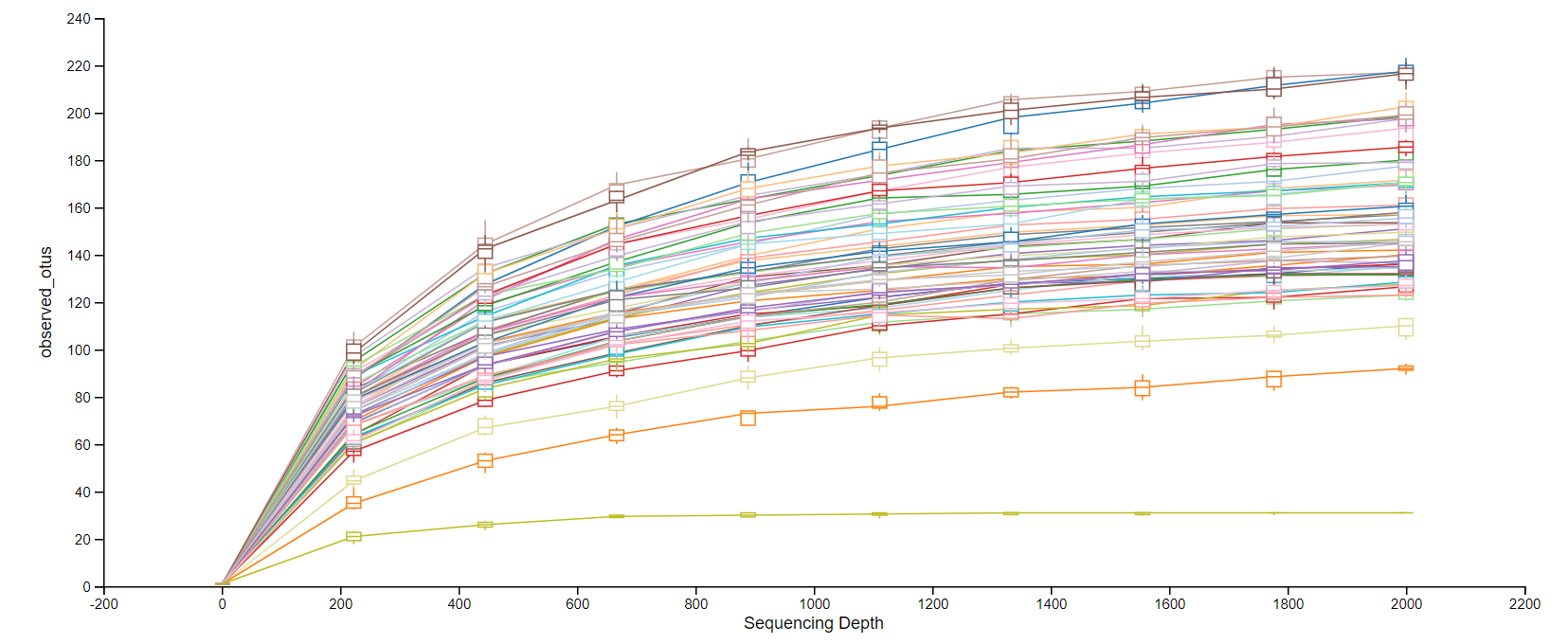


*Supplementary Table 1*, Sample demographics, including age class, social group, and habitat type

| Social group | N Male | N Female | N Juveniles | N Adults | Habitat Type | Percent Sampled (%) |
| --- | --- | --- | --- | --- | --- | --- |
| 10M | 1 | 1 | 0 | 2 | Remote | 75 |
| Bebedero | 2 | 3 | 1 | 4 | Village | 100 |
| Beti | 2 | 2 | 1 | 3 | Rural | 80 |
| Canal | 0 | 2 | 0 | 2 | Remote | 80 |
| Chupali | 1 | 4 | 1 | 4 | Village | 100 |
| Gonz | 0 | 1 | 0 | 1 | Remote | 80 |
| Hembra Negra | 1 | 4 | 0 | 5 | Rural | 80 |
| Huerta | 2 | 6 | 3 | 5 | Rural | 100 |
| IGN | 1 | 0 | 0 | 1 | Remote | 100 |
| Inchi | 2 | 2 | 1 | 3 | Village | 80 |
| Marisa | 0 | 1 | 0 | 1 | Village | 100 |
| Musculoso | 2 | 3 | 1 | 4 | Village | 80 |
| Otero | 2 | 2 | 1 | 3 | Village | 70 |
| Punta Sur | 1 | 1 | 0 | 2 | Remote | 75 |
| Sena | 2 | 3 | 1 | 4 | Rural | 100 |
| Tacuaral | 1 | 2 | 1 | 2 | Rural | 80 |
| Zunino | 1 | 2 | 0 | 3 | Rural | 100 |

*Supplementary Table 2*, PCR amplification conditions

| Gene | Fragment amplified | PCR conditions |
| --- | --- | --- |
| gdh | 599bp | Initial denaturation at 94C for 3 minutes; 35 cycles of denaturation at 94C for 45 seconds, annealing at 58C for 45 seconds, and extension at 72C for 1 minute; final extension at 72C for 10 minutes |
| tpi | 530bp | Initial denaturation at 95C for 2 minutes; 35 cycles of denaturation at 95C for 1 minutes, annealing for 45 seconds (54C and 58C for primary and nested reactions, respectively), and extension at 72C for 1 minute; final extension at 72C for 10 minutes. |
| bg | 511bp | Initial denaturation at 94C for 3 minutes; 35 cycles of denaturation at 94C for 20 seconds, annealing for 30 seconds (65C and 64C for primary and nested reactions, respectively), and extension at 72C for 1 minute; final extension at 72C for 10 minutes |

*Supplementary Table 3*, Bacterial taxa which are differentially abundant in uninfected individuals (LEfSe (LDA > 2)

| Betaproteobacteria | Barnesiellaceae |
| --- | --- |
| Flavobacterium succinicans | F16 (TM7) |
| Veillonellaceae | Anaeroplasma |
| Coriobacteriaceae | Blautia |
| TG5 | Victivalli vadensis |
| Ruminococcus gnavus | Butyricicoccus pullicaecorum |
| Butyricimonas | Ruminococcus |
| Mollicutes | Sharpea |
| Lachnospiraceae | Proteobacteria |
| Roseburia |  |
